# Self-Reference Effect Induced by Self-Cues Presented During Retrieval

**DOI:** 10.3389/fpsyg.2021.562359

**Published:** 2021-03-16

**Authors:** Liguo He, Wei Han, Zhan Shi

**Affiliations:** School of Psychology, Shenzhen University, Shenzhen, China

**Keywords:** self, memory, self-reference effect, encoding, retrieval

## Abstract

The self-reference effect (SRE) refers to better memory for self-relevant than for other-relevant information. Generally, the SRE is found in conditions in which links between the stimuli and the self are forged in the encoding phase. To investigate the possibility that such conditions are not prerequisites for the SRE, this research developed two conditions by using two recognition tasks involving abstract geometric shapes (AGSs). One was the cue-in-encoding condition in which self- and other-cues were presented to construct links with AGSs during the encoding phase, and the other was the cue-in-retrieval condition in which self- and other-cues were presented to construct links with AGSs during the retrieval phase. The SRE was found in both conditions. The findings reveal that self-cues merely presented during the retrieval phase are sufficient to induce the SRE. Links between the stimuli and the self constructed during the encoding phase may not be necessary prerequisites for the SRE.

## Introduction

The modulation of cognition and behavior by the self, a well-developed construct, has been verified by accumulating data (Cunningham and Turk, 2017). Most of these studies attempted to illustrate how the self influences memory. As the self is thought to originate from socially engineered mental schema of motives, emotions, actions, and outcomes of both oneself and others (Murray et al., 2014), these studies compared self- with other-referential processing and revealed a processing bias, termed the self-reference effect (SRE), toward self- rather than other-referential information (Klein, 2012). For example, when participants are required to report whether traits are descriptive of oneself or another person (e.g., “does this word describe you?”, “does this word describe Michael I. Posner?”), a memory advantage emerges for self-referential processing (Rogers et al., 1977). The SRE may be a measurement of a simple and basic distinction between self and non-self (Schäfer and Frings, 2019), which is called the minimal self (Gallagher, 2000). In traditional paradigms, the SRE was widely scrutinized and demonstrated in conditions in which the self was linked with stimuli by presenting self-cues during the encoding phase (Symons and Johnson, 1997).

The mechanisms of the SRE derived from these earlier studies are mixed (Legrand and Ruby, 2009; Gallagher, 2013). One line of research argued that the SRE is only a phenomenon concerning the level of processing and obeys the depth of processing theory (Craik and Tulving, 1975). For example, it has been suggested that the SRE may be attributed to superior elaborative and organizational properties of self-referential processing (Klein and Loftus, 1988). This mechanism implicates that it may be a prerequisite for the SRE to construct links between the stimuli and the self during the encoding phase. When stimuli and self-cues are encoded simultaneously, it is possible that the self may strengthen the level of processing of stimuli and then the SRE occurs. Objecting to simply using the levels of processing theory to interpret the SRE, another line of research argued that the SRE is a reflection of the functional distinction between self- and other-referential processing (Kelley et al., 2002). For example, the theory of cortical midline structures (CMS) has suggested that the CMS are the core mechanism of the SRE, which may distinguish all kinds of self- from other-referential information (Northoff et al., 2006). In terms of the view of the functional difference of the self, the SRE should not absolutely obey the depth of processing theory. If so, it is reasonable to conclude that the SRE may occur under conditions in which, on one hand, there are links between stimuli and self-cues, regardless of when and where the links are constructed, on the other hand, it is not necessary to construct the links between the stimuli and the self constructed during the encoding phase. For example, the SRE may emerge under contexts in which the links between the external stimuli and the self are constructed during the retrieval phase in everyday memory operations.

The present study aimed to examine whether the SRE might occur under such contexts. We compared memory performance in two incidental recognition tasks. In one, self- and other-cues were presented to construct links with stimuli during the encoding phase, whereas no self- and other-cues were presented during the retrieval phase. In the other, no self- and other-cues were presented during the encoding phase, whereas self- and other-cues were presented to construct links with the stimuli during the retrieval phase. The SRE was expected to emerge in the two conditions, respectively. Here, we name the condition in the former task the cue-in-encoding (CIE) condition and call the former SRE the encoding-SRE (E-SRE). We also name the condition in the latter task the cue-in-retrieval (CIR) condition and call the latter SRE the retrieval-SRE (R-SRE). In addition, to rule out the effects of familiarity or potential confounding cue–stimulus associations linked with previous experience that may provide a chance to give a high degree of importance to the self and a low degree of importance to other people, abstract geometric shapes (AGSs) were utilized as stimuli because they have few meanings and then are more difficult to forge associations with cues than with other stimuli, such as words, pictures, and objects.

## Materials and Methods

### Participants and Design

One hundred fifty-six undergraduates (74 females, mean age 19.07 years, range 18–21 years) participated in the present study. All participants were right-handed with normal or corrected-to-normal vision. The present study was approved by the local ethics committee and carried out in compliance with the Declaration of Helsinki. All participants gave written informed consents. The present study had a 2 (Cue: Self or Other) × 2 (Condition: CIE or CIR) mixed design, with Cue as the repeated measure.

### Materials and Procedure

Each participant was randomly assigned to either the CIE or CIR condition. Participants entered the laboratory with a confederate posing as a second participant (e.g., Mike was the participant and Joe was the confederate). The experimenter introduced them to each other and then asked them to participate in a game concerning AGSs. Before the game, each participant completed one of the two recognition tasks. The tasks including an encoding phase and a retrieval phase were presented on a PC using E-prime software (version 2.0, Psychology Software Tools).

**Figure 1:**
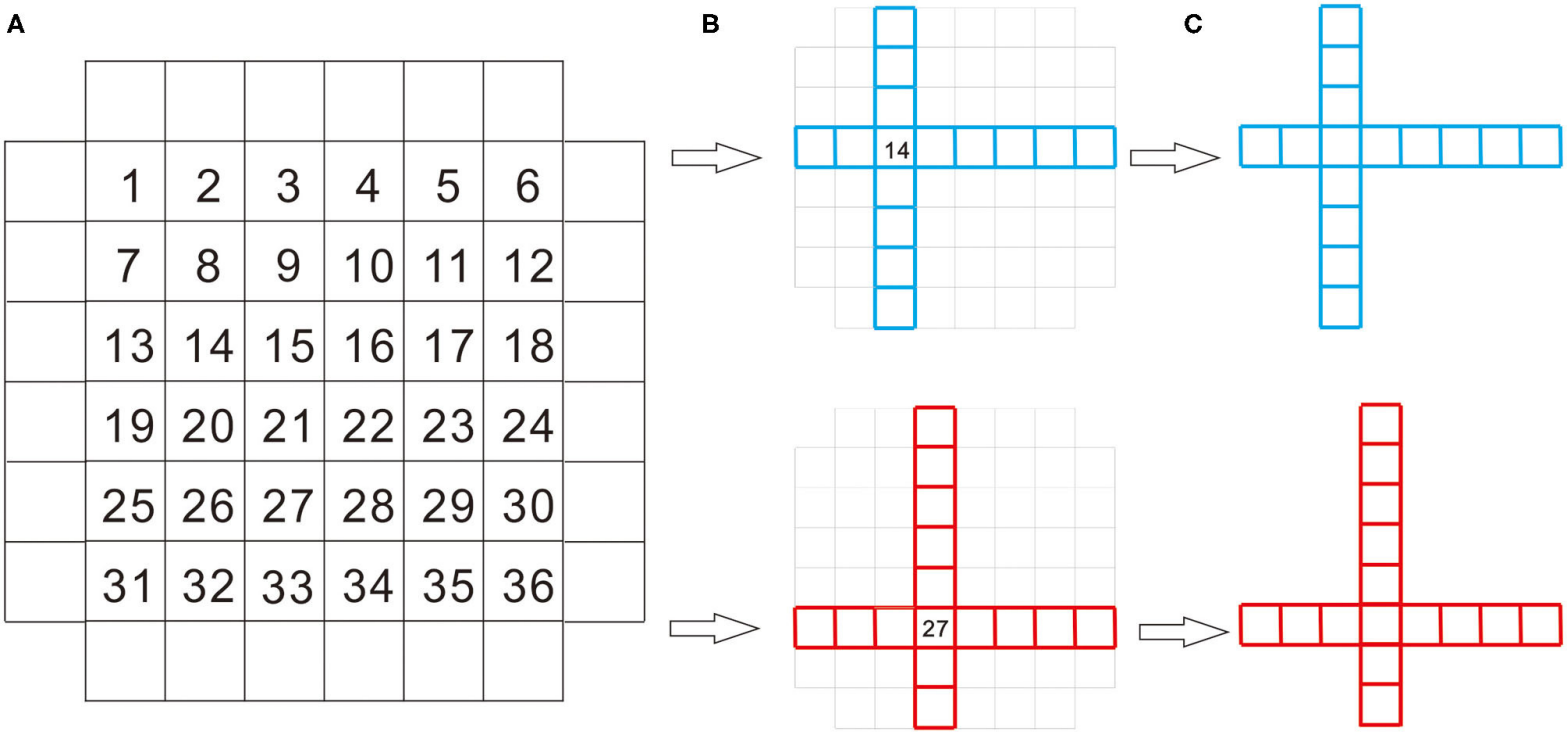
The process of stimulus formation. The stimuli used in the present study comprised a set of 36 abstract geometric shapes (AGSs). AGSs are composed of a series of squares arranged horizontally, plus a vertical series. There was always the same number of squares horizontally and vertically. The 36 numbers in **(A)** represent the space where the two series cross and 36 stimuli. AGSs differ by the number of squares below and above, as well as the left and right of the overlapping square **(B)**. Two of AGSs are shown as examples in **(C)**.

**Figure 2:**
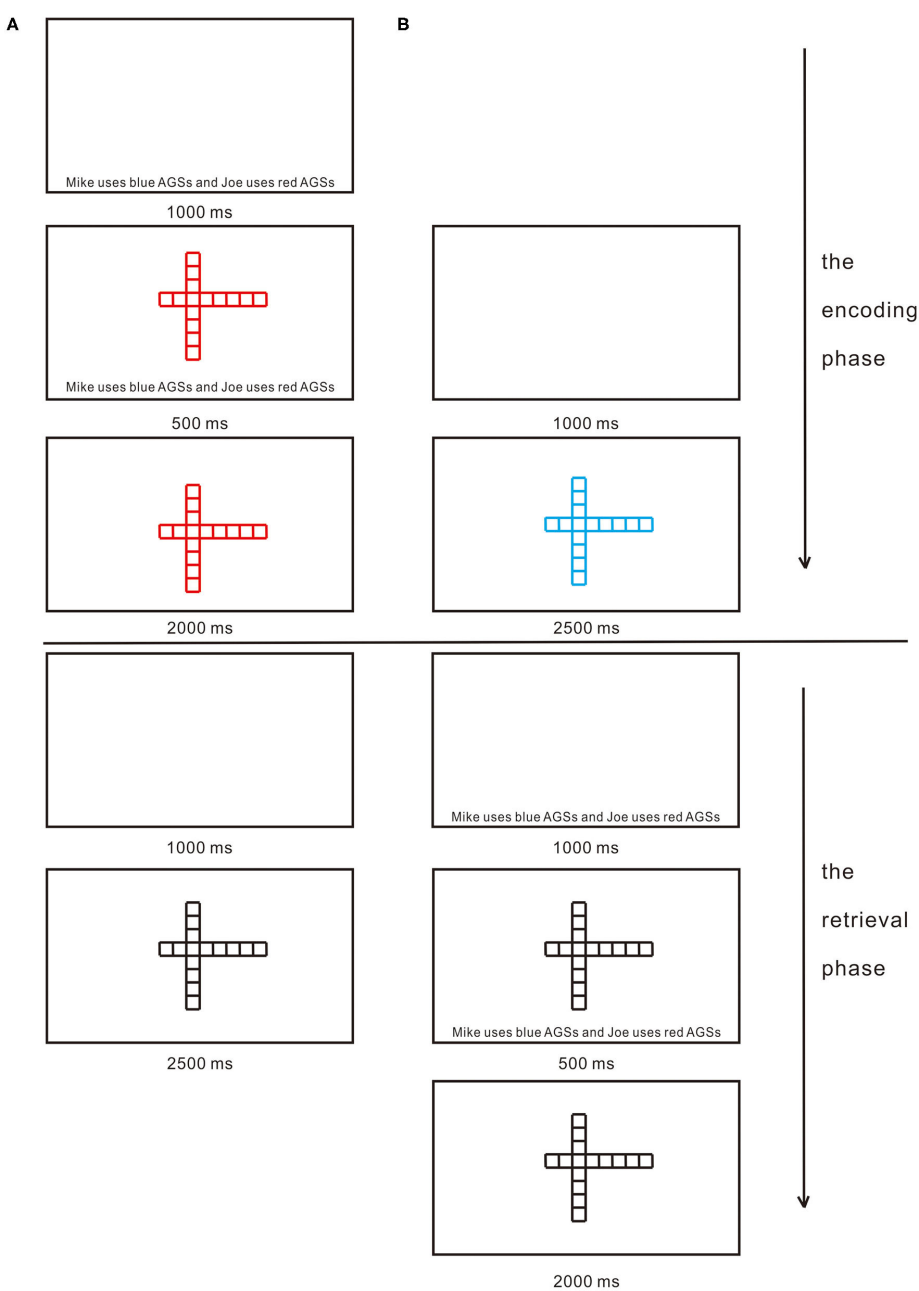
Timeline of stimuli. **(A)** The encoding and retrieval phases under the cue-in-encoding (CIE) condition. **(B)** The encoding and retrieval phases under the cue-in-retrieval (CIR) condition. For each participant, abstract geometric shapes (AGSs) were randomly divided into three equal lists. In the encoding phase in CIE and the encoding phase in CIR, AGSs from two of the lists (i.e., 24 AGSs) were presented with one list in blue and the other in red randomly. In the retrieval phase in CIE and the retrieval phase in CIR, AGSs from all three lists (i.e., two lists presented in the encoding phase and one list previously unseen) were randomly presented in black. In the encoding phase in CIE and the retrieval phase in CIR, half of the participants were reminded that they were to use blue AGSs, and the other half of the participants were reminded that they were to use red AGSs. For example, “Mike uses blue AGSs and Joe uses red AGSs” or “Mike uses red AGSs and Joe uses blue AGSs.” Each participant was randomly assigned to either using blue AGSs or using red AGSs.

In the encoding phase, each trial began with a blank screen for 1,000 ms, randomly followed by one of AGSs being presented in the center of the computer screen for 2,500 ms. A total of 36 AGSs were used in the present study. AGSs consist of two series of squares, with the same number of squares horizontally and vertically (see Figure 1). What makes the difference between the stimuli is the location of the intersection of the two series of squares. For each participant, AGSs were randomly divided into three equal lists. AGSs from two of the lists (i.e., 24 AGSs) were presented in the encoding phase with one list in blue and the other in red randomly. The third list was retained for use as foils in the subsequent memory test. Each participant was asked to judge what color each of AGSs was by key responses counterbalanced across participants. Only in the CIE condition, a sentence was presented in the blank screen for 1,000 ms as self- and other-cues reminding participants which color AGSs they were to use. Half of the participants were reminded that they were to use blue AGSs, and the other half of the participants were reminded that they were to use red AGSs. For example, “Mike uses blue AGSs and Joe uses red AGSs” or “Mike uses red AGSs and Joe uses blue AGSs.” Each participant was randomly assigned to either using blue AGSs or using red AGSs. Five hundred ms after the onset of AGSs, the sentence disappeared (see Figure 2). The encoding phase lasted about 2 min.

After a digit backward task (5 min), a surprise memory test (i.e., the retrieval phase) was administered concerning AGSs from all three lists (i.e., two lists presented in the encoding phase and one list previously unseen). Each test trial began with a blank screen for 1,000 ms, randomly followed by one of AGSs being presented in black in the center of the computer screen for 2,500 ms. Participants were instructed to complete “Old” or “New” judgments by key responses counterbalanced across participants. Only in the CIR condition, a sentence was presented in the blank screen for 1,000 ms as self- and other-cues reminding participants which color AGSs they were to use. Half of the participants were reminded that they were to use blue AGSs, and the other half of the participants were reminded that they were to use red AGSs. For example, “Mike uses blue AGSs and Joe uses red AGSs” or “Mike uses red AGSs and Joe uses blue AGSs.” Each participant was randomly assigned to either using blue AGSs or using red AGSs. Five hundred ms after the onset of AGSs, the sentence disappeared (see Figure 2). The AGSs were black at retrieval to make sure that the SRE could occur only through a retrospective link between the shape of the AGSs, the color of the AGSs, and the self (for details, see the Discussion section). After completion of the task, participants were thanked, debriefed, and dismissed.

## Results

**Figure 3:**
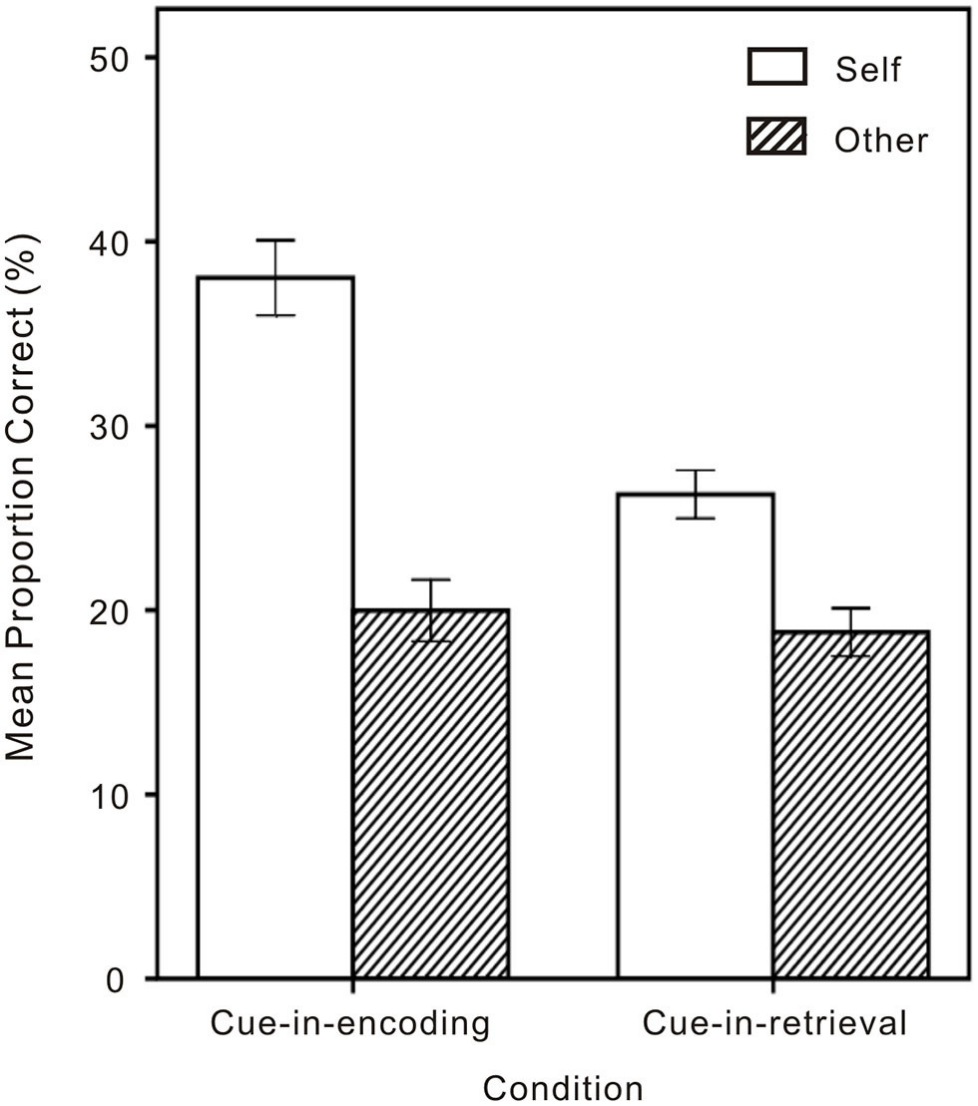
Memory performance on Self and Other trials in the cue-in-encoding and the cue-in-retrieval conditions. Error bars represent one standard error from the mean.

To evaluate the SRE, a mixed 2 (Cue: Self or Other) × 2 (Condition: CIE or CIR) analysis of variance (ANOVA) was performed on recognition memory data that were converted into proportional accuracy scores and corrected for guessing by subtracting the proportion of false alarms from the proportion of hits (Cunningham et al., 2008; Turk et al., 2008; Shi and He, 2020). The analysis revealed a significant interaction between Cue and Condition [*F*_(1,154)_ = 29.68, *p* < 0.001, η_*p*_^2^ = 0.16] (see Figure 3), with two main effects: for Cue, memory performance being significantly higher on Self than on Other trials [*F*_(1,154)_ = 172.97, *p* < 0.001, η_*p*_^2^ = 0.53; *Ms* = 0.32 vs. 0.19, respectively]; for Condition, memory performance being significantly higher for CIE than for CIR [*F*_(1,154)_ = 9.87, *p* = 0.002, η_*p*_^2^ = 0.06; *Ms* = 0.29 vs. 0.23, respectively].

The simple effects indicated that both memory performance of CIR and CIE were better on Self than on Other trials (*p* < 0.001 and *p* < 0.001, respectively), with memory performance of CIE being better than of CIR on Self trials (*p* < 0.001) but no difference between memory performance of CIE and CIR on Other trials (*p* = 0.581).

## Discussion

The present results reveal an SRE in both of the recognition tasks, that is, when a self- or other-relevant cue was presented at encoding or at retrieval. Moreover, the magnitude of the SRE was larger when self-relevant cue was presented at encoding than when it was presented at retrieval. Self-cues presented during both the encoding and retrieval phases can impact on memory performance. AGSs linked to self-cues presented during retrieval as well as during encoding were better remembered than those linked to other-cues. AGSs in the CIE condition were better remembered than those in the CIR condition.

In the CIE condition, self- and other- cues were presented during the encoding phase. This manipulation resulted in differences between the processing of the stimuli of Self and Other trials during both the encoding and retrieval phases. In the CIR condition, neither self-cues nor other-cues were presented during the encoding phase, and the two kinds of cues were presented during the retrieval phase. This manipulation did not result in differences between the processing of the stimuli of Self and Other trials during the encoding phase but resulted in differences between the stimuli of Self and Other trials during the retrieval phase. Self-cues merely presented during the retrieval phase were sufficient to induce the SRE. This means that the SRE can be induced by links between the processing of the self and stimuli, regardless of when and where the links are constructed. The emergence of the R-SRE provides a possibility to reconsider the contribution of links between the stimuli and the self constructed during the encoding phase to the SRE, which may not be necessary prerequisites for the SRE.

Moreover, the current results demonstrate the differences and relationship between the E-SRE and the R-SRE. Firstly, there were no differences between the impact of other-cues on the memory of stimuli under the CIE and CIR conditions. The results reveal that the R-SRE, as well as the E-SRE, is determined by self-cues but not by other-cues. Secondly, the influence of self-cues on stimuli during the encoding phase was larger than that during the retrieval phase. The findings raise the intriguing possibility that although both the E-SRE and the R-SRE may be reflections of the SRE, the two effects may reflect different mechanisms of the SRE, respectively. This conclusion is reasonable because AGSs were linked with self-cues during encoding in the CIE condition, whereas AGSs were linked with self-cues during retrieval in the CIR condition. It is the difference between encoding and retrieval that produces the difference between the E-SRE and the R-SRE. The encoding and retrieval phases are different components of memory, respectively. Hence, encoding and retrieval comprise two completely separate channels through which the self impacts on memory. However, it is also possible that the E-SRE and the R-SRE share a similar functional system specialized for self-processing: the mechanism concerning the functional difference of the self and others. This reasoning is consistent with Kelley et al.'s (2002) argument that the processing of self-referential information is not just “deeper” but is functionally distinct from other-referential processing.

Attention may be the key factor for the functional difference of the self and others in the present study. In an AGS, there are two types of information that can be memorized: the first type of information is the color (blue or red) and the second type of information is the shape. In the CIE, self- and other-cues were presented during the encoding phase, for example, “Mike uses blue AGSs and Joe uses red AGSs” or “Mike uses red AGSs and Joe uses blue AGSs.” Following the cue, an AGS was presented in blue or red. When processing the cue and an AGS presented in the screen, there should be three links forged during the encoding phase on the basis of the cue and the external representations of the color and the shape of an AGS. One link was forged between the color and the shape. Another link was forged between the color and self or another being. The third link was forged between the shape and self or another being, which was constructed on the basis of the former two links. Hence, according to the theory of Self-Attention Network (Humphreys and Sui, 2015), more attentional resources should be allocated to the shape linked with self relative to that linked with another being. During the retrieval phase, AGSs including the AGSs presented in blue or red during the encoding phase and the AGSs previously unseen were presented to participants in black. Participants were asked to complete “Old” or “New” judgments. All AGSs were presented in black to make sure that the recognition of an AGS depended on the shape presented in the retrieval phase and the three external representation-based links forged during the encoding phase. Consistent with a number of studies (see a review by Humphreys and Sui, 2015), more attentional resources were allocated to self-related shape that should be retrieved more easily than other-related shape, which was demonstrated in the present study.

In the CIR, no self- and other-cues but only the AGSs were presented in blue or red during the encoding phase. When processing an AGS presented in the screen, there should be only one external representation-based link between the color and the shape forged during the encoding phase. Hence, attention resources were evenly allocated to the shapes in blue and red. During the retrieval phase, self- and other-cues that were similar to those in the encoding phase in the CIE were presented. When processing the cue, there should be two links forged during the retrieval phase. One link was forged between self or another being and the internal representations of the colors. Another link was forged between self or another being and the internal representations of the shapes. Unlike the external representation-based links forged during the encoding phase in both CIE and CIR on the basis of the shapes or colors presented in the screen, the links forged during the retrieval phase in the CIR are on the basis of the internal representations of the shapes or colors. Previous research on memory retrieval suggested that some aspects of attention and memory might even reflect the same processes (Chun and Turk-Browne, 2007). Moreover, selective attention to internal representations may play an important role in memory retrieval (Badre et al., 2005; Wagner et al., 2005). Hence, similar to more attentional resources being allocated to the shapes (presented in the screen during the encoding phase in CIE) linked with self, more attentional resources were allocated to the internal representations of the shapes linked with self relative to those linked with another being during the retrieval phase. Following self- and other-cues, the AGSs that were similar to those in the retrieval phase in the CIE were presented. Participants were asked to complete “Old” or “New” judgments. All AGSs were presented in black to make sure that the recognition of an AGS depends on the shape presented in the retrieval phase, the external representation-based link forged during the encoding phase, and the two internal representation-based links forged during the retrieval phase. As more attentional resources were allocated to the internal representation of self-related shape, self-related shape should be retrieved more easily than other-related shape, which was also demonstrated in the present study. Hence, despite the difference between the E-SRE and the R-SRE, the functional difference between self- and other-referential information may be products of attention.

A recent theory of Binding and Retrieval in Action Control (BRAC) provides a framework for discussing the difference between the E-SRE and the R-SRE. Feature binding and retrieval are defined by BRAC as functionally different and separable processes that independently contribute to action-related phenomena (Frings et al., 2020). Features of stimuli are integrated or bound together. Repetition of any feature triggers the retrieval (Hommel et al., 2001; Frings et al., 2020). Recently, BRAC has been applied to interpret the effects on self-relevance. In research examining the effects of self-reference on stimulus processing, Schäfer et al. (2020) have argued that once stimuli are perceived as being relevant for our self, self-relevance serves to create a network of those contents and elements by binding them together, and the self would be represented by bindings of particular features. In the present study, it is a sentence “Mike uses blue AGSs and Joe uses red AGSs” that binds the features, such as the color and shapes of AGSs to the self or another person. Schäfer et al. (2020) also have argued that each feature can be weighted according to the current context and then feature weights influence stimulus processing. In the present study, different weights were given to self- vs. other-relevant features or features in CIE vs. CIR. The differences between the weights of self- vs. other-relevant features are the prerequisites of the E-SRE and the R-SRE. The interaction between the weights derived from 2 (Cue: Self or Other) × 2 (Condition: CIE or CIR) may be a possible mechanism to interpret the difference between the E-SRE and the R-SRE. Future research should be designed to further explore this issue under the guidance of BRAC.

One limitation of the present study concerns the AGSs having no real ecological value in the environment and being difficult to integrate into autobiographical memories that are by definition self-referential (Palombo et al., 2018). However, consistent with the present findings, previous studies have found that geometric shapes could be expanded to the self by tagging a neutral shape with a self-relevant label and could obtain behavioral self-bias (Sui et al., 2012, 2013). More importantly, the effect has meanwhile been replicated by other laboratories (Stein et al., 2016; Wozniak et al., 2018; Schäfer et al., 2020), in which the effects have been reasonably interpreted by using the BRAC framework (Schäfer et al., 2020).

Despite these unresolved issues, this study is the first to clearly demonstrate that the mere presence of a self cue during retrieval of AGSs is sufficient to enhance memory of AGSs linked with the self.

## Data Availability Statement

The original contributions presented in the study are included in the article/Supplementary Material, further inquiries can be directed to the corresponding author.

## Ethics Statement

The studies involving human participants were reviewed and approved by Ethics Committee of Shenzhen University. The patients/participants provided their written informed consent to participate in this study.

## Author Contributions

LH designed and executed the study, and wrote the paper. WH executed the study and analyzed the data. ZS designed the study, analyzed the data, and wrote the paper. All authors approved the final version of the manuscript for submission.

## Conflict of Interest

The authors declare that the research was conducted in the absence of any commercial or financial relationships that could be construed as a potential conflict of interest.

## References

[B1] Badre D. Poldrack R. A. Paré-Blagoev E. J. Insler R. Z. Wagner A. D. (2005). Dissociable controlled retrieval and generalized selection mechanisms in ventrolateral prefrontal cortex. Neuron 47, 907–918. 10.1016/j.neuron.2005.07.023 16157284

[B2] Chun M. M. Turk-Browne N. B. (2007). Interactions between attention and memory. Curr. Opin. Neurobiol. 17, 177–184. 10.1016/j.conb.2007.03.005 17379501

[B3] Craik F. I. Tulving E. (1975). Depth of processing and the retention of words in episodic memory. J. Exp. Psychol. Gen. 104, 268–294. 10.1037/0096-3445.104.3.268

[B4] Cunningham S. J. Turk D. J. (2017). A review of self- processing biases in cognition. QJ Exp. Psychol. 70, 987–995. 10.1080/17470218.2016.1276609 28059625

[B5] Cunningham S. J. Turk D. J. MacDonald L. M. Macrae C. N. (2008). Yours or mine? Ownership and memory. Conscious. Cogn. 17, 312–318. 10.1016/j.concog.2007.04.003 17543540

[B6] Frings C. Hommel B. Koch I. Rothermund K. Dignath D.. (2020). Binding and Retrieval in Action Control (BRAC). Trends Cogn. Sci. (Regul. Ed) 24, 375–387. 10.1016/j.tics.2020.02.004 32298623

[B7] Gallagher S. (2000). Philosophical conceptions of the self: implications for cognitive science. Trends Cogn. Sci. (Regul. Ed) 4, 14–21. 10.1016/S1364-6613(99)01417-5 10637618

[B8] Gallagher S. (2013). A pattern theory of self. Front. Hum. Neurosci. 7:443. 10.3389/fnhum.2013.00443 23914173 PMC3730118

[B9] Hommel B. Müsseler J. Aschersleben G. Prinz W. (2001). The theory of event coding (TEC): a framework for perception and action planning. Behav. Brain Sci. 24, 849–878. 10.1017/S0140525X01000103 12239891

[B10] Humphreys G. W. Sui J. (2015). Attentional control and the self: the Self-Attention Network (SAN). Cogn. Neurosci. 7, 5–17. 10.1080/17588928.2015.1044427 25945926

[B11] Kelley W. M. Macrae C. N. Wyland C. L. Caglar S. Inati S. Heatherton T. F. (2002). Finding the self? An event-related fMRI study. J. Cogn. Neurosci. 14, 785–794. 10.1162/08989290260138672 12167262

[B12] Klein S. B. (2012). Self, memory, and the self-reference effect: an examination of conceptual and methodological issues. Pers. Soc. Psychol. Rev. 16, 283–300. 10.1177/1088868311434214 22291045

[B13] Klein S. B. Loftus J. (1988). The nature of self-referent encoding: the contributions of elaborative and organizational processes. J. Pers. Soc. Psychol. 55, 5–11. 10.1037/0022-3514.55.1.5

[B14] Legrand D. Ruby P. (2009). What is self-specific? Theoretical investigation and critical review of neuroimaging results. Psychol. Rev. 116:252. 10.1037/a0014172 19159156

[B15] Murray R. J. Debbane M. Fox P. T. Bzdok D. Eickhoff S. B. (2014). Functional connectivity mapping of regions associated with self- and other-processing. Hum. Brain Mapp. 36, 1304–1324. 10.1002/hbm.22703 25482016 PMC4791034

[B16] Northoff G. Heinzel A. De Greck M. Bermpohl F. Dobrowolny H. Panksepp J. (2006). Self-referentialprocessing in our brain—a meta-analysis of imaging studies on the self. Neuroimage 31, 440–457. 10.1016/j.neuroimage.2005.12.002 16466680

[B17] Palombo D. J. Sheldon S. Levine B. (2018). Individual differences in autobiographical memory. Trends Cogn. Sci. (Regul. Ed) 22, 583–597. 10.1016/j.tics.2018.04.007 29807853

[B18] Rogers T. B. Kuiper N. A. Kirker W. S. (1977). Self-reference and the encoding of personal information. J. Pers. Soc. Psychol. 35, 677–688. 10.1037/0022-3514.35.9.677 909043

[B19] Schäfer S. Frings C. (2019). Understanding self-prioritisation: the prioritisation of self-relevant stimuli and its relation to the individual self-esteem. J. Cogn. Psychol. 31, 813–824. 10.1080/20445911.2019.1686393

[B20] Schäfer S. Wentura D. Frings C. (2020). Creating a network of importance: The particular effects of self-relevance on stimulus processing. Atten. Percept. Psycho. 82, 3750–3766. 10.3758/s13414-020-02070-7 32557005 PMC7536139

[B21] Shi Z. He L. (2020). Mindfulness: attenuating self-Referential processing and strengthening other-Referential processing. Mindfulness (N. Y) 11, 59–605. 10.1007/s12671-019-01271-y

[B22] Stein T. Siebold A. Zoest W. V. (2016). Testing the idea of privileged awareness of self-relevant information. J. Exp. Psychol. Hum. Percept. Perform. 42, 303–307. 10.1037/xhp0000197 26727020

[B23] Sui J. He X. Humphreys G. W. (2012). Perceptual effects of social salience: evidence from self-prioritization effects on perceptual matching. J. Exp. Psychol. Hum. Percept. Perform. 38, 1105–1117. 10.1037/a0029792 22963229

[B24] Sui J. Rotshtein P. Humphreys G. W. (2013). Coupling social attention to the self forms a network for personal significance. PNAS 110, 7607–7612. 10.1073/pnas.1221862110 23610386 PMC3651422

[B25] Symons C. S. Johnson B. T. (1997). The self-reference effect in memory: a meta-analysis. Psychol. Bull. 121, 371–394. 10.1037/0033-2909.121.3.371 9136641

[B26] Turk D. J. Cunningham S. J. Macrae C. N. (2008). Self-memory biases in explicit and incidental encoding of trait adjectives. Conscious. Cogn. 17, 1040–1045. 10.1016/j.concog.2008.02.004 18395467

[B27] Wagner A. D. Shannon B. J. Kahn I. Buckner R. L. (2005). Parietal lobe contributions to episodic memory retrieval. Trends Cogn. Sci. (Regul. Ed) 9, 445–453. 10.1016/j.tics.2005.07.001 16054861

[B28] Wozniak M. Kourtis D. Knoblich G. (2018). Prioritization of arbitrary faces associated to self: an EEG study. PLoS ONE 13:e0190679. 10.1371/journal.pone.0190679 29293670 PMC5749812

