# Supplementary material for: Self-Reference Effect Induced by Self-Cues Presented During Retrieval
Source: Front Psychol. 2021 Mar 16;12:562359. doi: 10.3389/fpsyg.2021.562359 (PMC8007774; doi:10.3389/fpsyg.2021.562359)
Supplement: Supplementary file 1 [file Data_Sheet_1.DOC]

# Supplementary Data

| Subjects | Condition | Self | Other |
| --- | --- | --- | --- |
| S01 | Cue-in-encoding | 0.333333333 | 0.25 |
| S02 | Cue-in-encoding | 0.5 | 0.166666667 |
| S03 | Cue-in-encoding | 0.25 | 0.083333333 |
| S04 | Cue-in-encoding | 0.416666667 | 0.083333333 |
| S05 | Cue-in-encoding | 0.666666667 | 0.583333333 |
| S06 | Cue-in-encoding | 0.416666667 | 0.333333333 |
| S07 | Cue-in-encoding | 0.166666667 | 0.083333333 |
| S08 | Cue-in-encoding | 0.666666667 | 0.083333333 |
| S09 | Cue-in-encoding | 0.166666667 | 0.083333333 |
| S10 | Cue-in-encoding | 0.25 | 0.083333333 |
| S11 | Cue-in-encoding | 0.166666667 | 0.083333333 |
| S12 | Cue-in-encoding | 0.583333333 | 0.083333333 |
| S13 | Cue-in-encoding | 0.25 | 0.166666667 |
| S14 | Cue-in-encoding | 0.333333333 | 0.25 |
| S15 | Cue-in-encoding | 0.25 | 0.083333333 |
| S16 | Cue-in-encoding | 0.166666667 | 0.083333333 |
| S17 | Cue-in-encoding | 0.666666667 | 0.083333333 |
| S18 | Cue-in-encoding | 0.166666667 | 0.083333333 |
| S19 | Cue-in-encoding | 0.25 | 0.333333333 |
| S20 | Cue-in-encoding | 0.333333333 | 0.25 |
| S21 | Cue-in-encoding | 0.416666667 | 0.416666667 |
| S22 | Cue-in-encoding | 0.166666667 | 0.083333333 |
| S23 | Cue-in-encoding | 0.166666667 | 0.083333333 |
| S24 | Cue-in-encoding | 0.666666667 | 0.083333333 |
| S25 | Cue-in-encoding | 0.416666667 | 0.083333333 |
| S26 | Cue-in-encoding | 0.25 | 0.083333333 |
| S27 | Cue-in-encoding | 0.5 | 0.166666667 |
| S28 | Cue-in-encoding | 0.25 | 0.333333333 |
| S29 | Cue-in-encoding | 0.25 | 0.166666667 |
| S30 | Cue-in-encoding | 0.666666667 | 0.583333333 |
| S31 | Cue-in-encoding | 0.666666667 | 0.5 |
| S32 | Cue-in-encoding | 0.333333333 | 0.25 |
| S33 | Cue-in-encoding | 0.583333333 | 0.083333333 |
| S34 | Cue-in-encoding | 0.25 | 0.083333333 |
| S35 | Cue-in-encoding | 0.583333333 | 0.166666667 |
| S36 | Cue-in-encoding | 0.25 | 0.166666667 |
| S37 | Cue-in-encoding | 0.333333333 | 0.083333333 |
| S38 | Cue-in-encoding | 0.583333333 | 0.333333333 |
| S39 | Cue-in-encoding | 0.666666667 | 0.083333333 |
| S40 | Cue-in-encoding | 0.166666667 | 0.083333333 |
| S41 | Cue-in-encoding | 0.583333333 | 0.5 |
| S42 | Cue-in-encoding | 0.25 | 0.166666667 |
| S43 | Cue-in-encoding | 0.25 | 0.083333333 |
| S44 | Cue-in-encoding | 0.333333333 | 0.083333333 |
| S45 | Cue-in-encoding | 0.583333333 | 0.416666667 |
| S46 | Cue-in-encoding | 0.333333333 | 0.166666667 |
| S47 | Cue-in-encoding | 0.25 | 0.166666667 |
| S48 | Cue-in-encoding | 0.166666667 | 0.083333333 |
| S49 | Cue-in-encoding | 0.583333333 | 0.083333333 |
| S50 | Cue-in-encoding | 0.25 | 0.166666667 |
| S51 | Cue-in-encoding | 0.333333333 | 0.25 |
| S52 | Cue-in-encoding | 0.583333333 | 0.25 |
| S53 | Cue-in-encoding | 0.416666667 | 0.333333333 |
| S54 | Cue-in-encoding | 0.583333333 | 0.5 |
| S55 | Cue-in-encoding | 0.166666667 | 0.333333333 |
| S56 | Cue-in-encoding | 0.583333333 | 0.166666667 |
| S57 | Cue-in-encoding | 0.25 | 0.333333333 |
| S58 | Cue-in-encoding | 0.333333333 | 0.083333333 |
| S59 | Cue-in-encoding | 0.166666667 | 0.083333333 |
| S60 | Cue-in-encoding | 0.166666667 | 0.083333333 |
| S61 | Cue-in-encoding | 0.5 | 0.166666667 |
| S62 | Cue-in-encoding | 0.166666667 | 0.083333333 |
| S63 | Cue-in-encoding | 0.583333333 | 0.166666667 |
| S64 | Cue-in-encoding | 0.25 | 0.166666667 |
| S65 | Cue-in-encoding | 0.416666667 | 0.083333333 |
| S66 | Cue-in-encoding | 0.333333333 | 0.166666667 |
| S67 | Cue-in-encoding | 0.666666667 | 0.166666667 |
| S68 | Cue-in-encoding | 0.416666667 | 0.166666667 |
| S69 | Cue-in-encoding | 0.75 | 0.666666667 |
| S70 | Cue-in-encoding | 0.5 | 0.25 |
| S71 | Cue-in-encoding | 0.75 | 0.583333333 |
| S72 | Cue-in-encoding | 0.166666667 | 0.083333333 |
| S73 | Cue-in-encoding | 0.25 | 0.166666667 |
| S74 | Cue-in-encoding | 0.25 | 0.166666667 |
| S75 | Cue-in-encoding | 0.166666667 | 0.083333333 |
| S76 | Cue-in-encoding | 0.333333333 | 0.166666667 |
| S77 | Cue-in-encoding | 0.5 | 0.416666667 |
| S78 | Cue-in-encoding | 0.333333333 | 0.25 |
| S79 | Cue-in-retrieval | 0.333333333 | 0.25 |
| S80 | Cue-in-retrieval | 0.333333333 | 0.25 |
| S81 | Cue-in-retrieval | 0.166666667 | 0.083333333 |
| S82 | Cue-in-retrieval | 0.666666667 | 0.583333333 |
| S83 | Cue-in-retrieval | 0.333333333 | 0.25 |
| S84 | Cue-in-retrieval | 0.166666667 | 0.083333333 |
| S85 | Cue-in-retrieval | 0.5 | 0.416666667 |
| S86 | Cue-in-retrieval | 0.166666667 | 0.083333333 |
| S87 | Cue-in-retrieval | 0.5 | 0.416666667 |
| S88 | Cue-in-retrieval | 0.333333333 | 0.25 |
| S89 | Cue-in-retrieval | 0.25 | 0.166666667 |
| S90 | Cue-in-retrieval | 0.166666667 | 0.083333333 |
| S91 | Cue-in-retrieval | 0.416666667 | 0.333333333 |
| S92 | Cue-in-retrieval | 0.25 | 0.166666667 |
| S93 | Cue-in-retrieval | 0.333333333 | 0.25 |
| S94 | Cue-in-retrieval | 0.25 | 0.166666667 |
| S95 | Cue-in-retrieval | 0.166666667 | 0.083333333 |
| S96 | Cue-in-retrieval | 0.166666667 | 0.083333333 |
| S97 | Cue-in-retrieval | 0.25 | 0.166666667 |
| S98 | Cue-in-retrieval | 0.416666667 | 0.333333333 |
| S99 | Cue-in-retrieval | 0.166666667 | 0.166666667 |
| S100 | Cue-in-retrieval | 0.166666667 | 0.083333333 |
| S101 | Cue-in-retrieval | 0.583333333 | 0.5 |
| S102 | Cue-in-retrieval | 0.333333333 | 0.25 |
| S103 | Cue-in-retrieval | 0.166666667 | 0.083333333 |
| S104 | Cue-in-retrieval | 0.25 | 0.166666667 |
| S105 | Cue-in-retrieval | 0.166666667 | 0.083333333 |
| S106 | Cue-in-retrieval | 0.416666667 | 0.333333333 |
| S107 | Cue-in-retrieval | 0.25 | 0.166666667 |
| S108 | Cue-in-retrieval | 0.166666667 | 0.083333333 |
| S109 | Cue-in-retrieval | 0.25 | 0.166666667 |
| S110 | Cue-in-retrieval | 0.166666667 | 0.083333333 |
| S111 | Cue-in-retrieval | 0.25 | 0.166666667 |
| S112 | Cue-in-retrieval | 0.25 | 0.166666667 |
| S113 | Cue-in-retrieval | 0.166666667 | 0.083333333 |
| S114 | Cue-in-retrieval | 0.5 | 0.416666667 |
| S115 | Cue-in-retrieval | 0.416666667 | 0.333333333 |
| S116 | Cue-in-retrieval | 0.25 | 0.166666667 |
| S117 | Cue-in-retrieval | 0.333333333 | 0.25 |
| S118 | Cue-in-retrieval | 0.333333333 | 0.25 |
| S119 | Cue-in-retrieval | 0.166666667 | 0.083333333 |
| S120 | Cue-in-retrieval | 0.25 | 0.166666667 |
| S121 | Cue-in-retrieval | 0.416666667 | 0.333333333 |
| S122 | Cue-in-retrieval | 0.416666667 | 0.333333333 |
| S123 | Cue-in-retrieval | 0.333333333 | 0.25 |
| S124 | Cue-in-retrieval | 0.5 | 0.416666667 |
| S125 | Cue-in-retrieval | 0.166666667 | 0.083333333 |
| S126 | Cue-in-retrieval | 0.25 | 0.166666667 |
| S127 | Cue-in-retrieval | 0.333333333 | 0.25 |
| S128 | Cue-in-retrieval | 0.25 | 0.166666667 |
| S129 | Cue-in-retrieval | 0.166666667 | 0.083333333 |
| S130 | Cue-in-retrieval | 0.333333333 | 0.25 |
| S131 | Cue-in-retrieval | 0.166666667 | 0.083333333 |
| S132 | Cue-in-retrieval | 0.166666667 | 0.083333333 |
| S133 | Cue-in-retrieval | 0.25 | 0.166666667 |
| S134 | Cue-in-retrieval | 0.166666667 | 0.083333333 |
| S135 | Cue-in-retrieval | 0.083333333 | 0.083333333 |
| S136 | Cue-in-retrieval | 0.166666667 | 0.083333333 |
| S137 | Cue-in-retrieval | 0.25 | 0.166666667 |
| S138 | Cue-in-retrieval | 0.166666667 | 0.083333333 |
| S139 | Cue-in-retrieval | 0.166666667 | 0.416666667 |
| S140 | Cue-in-retrieval | 0.25 | 0.166666667 |
| S141 | Cue-in-retrieval | 0.25 | 0.166666667 |
| S142 | Cue-in-retrieval | 0.25 | 0.166666667 |
| S143 | Cue-in-retrieval | 0.25 | 0.166666667 |
| S144 | Cue-in-retrieval | 0.166666667 | 0.083333333 |
| S145 | Cue-in-retrieval | 0.25 | 0.166666667 |
| S146 | Cue-in-retrieval | 0.083333333 | 0.083333333 |
| S147 | Cue-in-retrieval | 0.166666667 | 0.083333333 |
| S148 | Cue-in-retrieval | 0.166666667 | 0.083333333 |
| S149 | Cue-in-retrieval | 0.25 | 0.166666667 |
| S150 | Cue-in-retrieval | 0.166666667 | 0.083333333 |
| S151 | Cue-in-retrieval | 0.25 | 0.166666667 |
| S152 | Cue-in-retrieval | 0.333333333 | 0.25 |
| S153 | Cue-in-retrieval | 0.166666667 | 0.083333333 |
| S154 | Cue-in-retrieval | 0.25 | 0.25 |
| S155 | Cue-in-retrieval | 0.166666667 | 0.083333333 |
| S156 | Cue-in-retrieval | 0.166666667 | 0.083333333 |
